# Diffusion of Single-Pass Transmembrane Receptors: From the Plasma Membrane into Giant Liposomes

**DOI:** 10.1007/s00232-016-9936-8

**Published:** 2016-11-08

**Authors:** Remigiusz Worch, Zdenek Petrášek, Petra Schwille, Thomas Weidemann

**Affiliations:** 1 Institute of Physics, Polish Academy of Sciences, Al. Lotników 32/46, 02-668, Warsaw, Poland; 2 Institute of Biotechnology and Biochemical Engineering, Graz University of Technology, Petersgasse 10-12/I, 8010, Graz, Austria; 3 Cellular and Molecular Biophysics, Max Planck Institute of Biochemistry, Am Klopferspitz 18, 82152 Martinsried, Germany

**Keywords:** Diffusion coefficient, Fluorescent lipid, Interleukin-4 receptor, Giant plasma membrane vesicles, Giant unilamellar vesicles, Fluorescence correlation spectroscopy

## Abstract

To quantitatively examine the effect of membrane organization on lateral diffusion, we studied fluorescent carbocyanine lipid analogues and EGFP-tagged, single-pass transmembrane proteins in systems of decreasing complexity: (i) the plasma membrane (PM) of living cells, (ii) paraformaldehyde/dithiothreitol-induced giant plasma membrane vesicles (GPMVs), and (iii) giant unilamellar vesicles (GUVs) under physiological buffer conditions. A truncated, signaling-deficient interleukin-4 receptor subunit, showing efficient accumulation in the plasma membrane, served as a model transmembrane protein. Two-dimensional diffusion coefficients (*D*) were determined by fluorescence correlation spectroscopy (FCS) either at fixed positions (single-point, spFCS) or while scanning a circular orbit (circular scanning, csFCS). Consistent with a different inclusion sizes in the membrane, lipids diffuse slightly faster than the single-spanning membrane proteins in both membrane systems, GUVs and GPMVs. In GPMVs lipids and proteins consistently experienced a fivefold larger viscosity than in GUVs, reflecting the significant fraction of plasma membrane-derived proteins partitioning into GPMVs. Lipid and protein diffusion in the PM was, respectively, 2 times and 4–5 times slower in comparison to GPMVs. This discrepancy was quantitatively confirmed by csFCS. The similarity of diffusion of receptors and lipids in GPMVs and GUVs and its significant difference in the plasma membrane suggest that protein domains as small as EGFP convey sensitivity to the actin cortex on various length scales.

## Introduction

Signal transduction at the cell surface requires that receptors adopt defined on- and off-states in accordance with ligand binding. Receptor activation can involve two distinct structural levels: ligand-induced conformational changes that propagate directly through the membrane to the cytosolic domains (tertiary structure) and ligand-induced binding or dissociation of receptor subunits within the membrane plane (quaternary structure). In many cases, receptor activation mechanisms represent a combination of both as exemplified by the seven-pass transmembrane G-protein-coupled receptors (GPCRs), common to all eukaryotes, where the ligand-induced conformational change in the transmembrane domain triggers dissociation of the cytosolic trimeric complex. However, due to the substantial complexity of dynamic processes simultaneously taking place in the native plasma membrane, many of the receptor activation mechanisms are not fully understood.

Taking a bioinformatics approach, we recently proposed that ligand-induced, diffusion-controlled receptor oligomerization gained increasing importance as a mode of signal transduction during later stages of metazoan evolution (Worch et al. 2010). We observed a raise in the number of genes coding for single-pass transmembrane proteins, reaching up to ~50% with respect to the total number of transmembrane proteins in higher eukaryotes. Because a single-membrane-spanning alpha-helix bears only a limited conformational space in which stable on- and off-states could evolve, it is conceivable that the expansion of single-pass transmembrane receptor subfamilies depended critically on combinatorial receptor compositions.

This evolutionary trend is recapitulated by the hematopoietic superfamily of mammalian cytokine receptors consisting entirely of single-pass transmembrane receptors: members of a small prototypic subgroup (e.g., erythropoietin receptor and growth hormone receptor), which are structurally closest to the precursor receptors in lower vertebrates, are expressed at the cell surface as pre-formed homodimers that mediate signaling by a conformational change (Brown et al. 2005; Remy et al. 1999). In contrast, evolutionary younger receptor subgroups feature extensive diversification through heterologous binding modes with variable stoichiometry (dimers, trimers, and hexamers), and the emergence of shared receptor subunits (Interleukin-2 receptor gamma, Interleukin-3 receptor beta, and glycoprotein 130; reviewed in Weidemann et al. 2007). Thus, to assess, the mobility of receptor subunits in the plasma membrane is of fundamental importance for a quantitative understanding of many signaling pathways, and in particular the hematopoietic cytokine receptors.

Although modern microscopy methods allow to perform mobility measurements at various length and time scales (Eggeling et al. 2009), the native plasma membrane bears conceptual complications for the interpretation of such data. Recent years have substantially changed the picture of the plasma membrane from the homogenous fluid mosaic model to a highly dynamic protein–lipid composite material. Growing evidence highlights the importance of the membrane lateral heterogeneity in cellular functions (Jacobson et al. 2007). One central hypothesis states that the plasma membrane contains ‘lipid rafts,’ nanometer-sized, cohesive domains enriched in cholesterol, sphingomyelin, and specific raft marker proteins (Simons and Ikonen 1997). In addition, the lateral organization of the plasma membrane is determined by the presence of a membrane-attached ‘cortex’ of cytoskeletal proteins (spectrin, actin, etc.). This cortex is believed to create a fence-like substructure of diffusion barriers (Kusumi et al. 2011), which became initially apparent as the so-called hop-diffusion (Fujiwara et al. 2002). While the physical principles governing hierarchical organization of the membrane are still under debate, it is widely accepted that lateral membrane heterogeneity is biologically significant and takes part in sorting and signaling from the surface (Le Roy and Wrana 2005; Lingwood and Simons 2010; Simons and Gerl 2010).

In general, lateral mobility of a molecule in the membrane can be either attributed to membrane properties of surrounding lipids or the molecule itself. Thus, a single-pass transmembrane construct qualifies as a model protein only in a strict absence of any lateral interactions, ideally showing a mono-disperse distribution in the membrane. We found a previously well-characterized C-terminally truncated and EGFP-tagged version of the interleukin-4 receptor alpha chain (IL-4Rα) suitable for this purpose (Hintersteiner et al. 2008; Worch et al. 2010). IL-4R is the rare example of a class I cytokine receptor for which ligand-induced heterodimerization was demonstrated in living cells at the single-molecule level by both, FCS as well as single-particle tracking (Gandhi et al. 2014; Moraga et al. 2015). Important to this study, a ligand-induced activation mechanism of IL-4R suggests that the individual subunits have evolved as non-interacting, freely diffusing entities, which was experimentally confirmed (Worch et al. 2010). Because the cytoplasmic tail of IL-4Rα is removed, the receptors are signaling-deficient but still functional in their competitive activity toward endogenous signaling components (Weidemann et al. 2011). We selected two mutant receptor versions that are distinguished solely by the absence or presence of a binding site for JAK1, the associated kinase that plays a role for trafficking and sorting along the constitutive secretory pathway (Radtke et al. 2002).

To dissect the environmental cues that govern lateral diffusion at the cell surface requires a gradual comparison with artificial membrane systems in which some defined aspects of the native plasma membrane are absent. One straight forward approach is to induce giant plasma membrane vesicles (GPMVs) directly from culture cells expressing the receptor constructs (Tank et al. 1982). These large, 10–50 µm sized, spherical vesicles bud off at the cell surface under a mildly toxic paraformaldehyde/dithiothreitol treatment by a mechanism resembling apoptosis (Keller et al. 2009). Mobile components of the plasma membrane but not cytoskeleton bound material are in diffusive continuity with the GPMV membrane. Devoid of the actin cortex, GPMVs are a low-noise platform to measure lateral diffusion in the absence of membrane trafficking while keeping the compositional complexity close to native. Many studies employed GPMVs to study protein partitioning into phase-separated lipid domains (Baumgart et al. 2003, 2007; Veatch et al. 2008). However, only a few reports addressed the diffusion of transmembrane proteins by FCS (Sezgin et al. 2012; Worch et al. 2010).

The chemical complexity of the membrane can be further reduced by reconstitution of the model receptors in giant unilamellar vesicles (GUVs). Due to their large size, GUVs are amenable to the most microscopic biophysical methods and are widely employed to study phase separation, unmixing and diffusion of fluorescent lipids and membrane proteins (Kahya 2010). In GUVs the lateral diffusion is largely dominated by the lipid composition, rather than the protein content. Although it is relatively simple to produce GUVs from pure lipids, single-pass transmembrane proteins seem to represent difficult candidates for reconstitution (Bacia et al. 2004; Kalvodova et al. 2005; Ramadurai et al. 2009; Streicher et al. 2009). Solubilization with detergents, partial drying, and higher temperatures during the sample preparation all can potentially degrade the protein. Moreover, the electroswelling procedure for GUV formation is limited to low, non-physiological salt conditions (~10 mM), thus far from physiological conditions (Pott et al. 2008; Shaklee et al. 2010). We therefore developed a combined two-step purification protocol that involves the isolation of receptor containing nanopatches (Swift et al. 2007, 2009) followed by GUV preparation under physiological buffer conditions using the agarose swelling method (Horger et al. 2009).

**Fig. 1:**
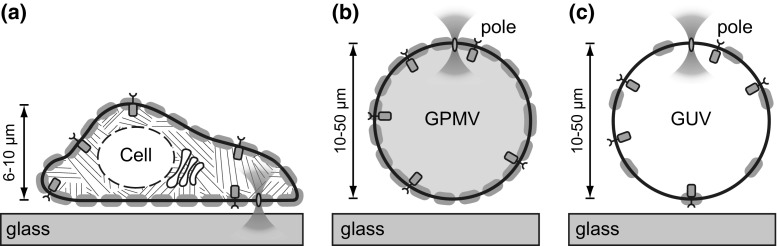
Experimental approach of measuring receptor diffusion in different types of membranes by confocal FCS. **a** Schematic *x*–*z* cross-section of a cell. The mobility of lipids and receptors was probed in the plasma membrane, where the lateral mobility is influenced by the viscosity of the lipid bilayer (*black thick line*), membrane associated proteins (*gray ovals*), and cytoskeleton (hatched). **b** Corresponding measurements at the poles of giant plasma membrane vesicles (GPMV) or **c** giant unilamellar vesicles (GUV). Note that GPMVs are partially filled with cytoplasmic protein (*gray shading*) and that the membrane protein content (*gray ovals*) decreases subsequently from **a** to **c**

In this study we systematically compare diffusion coefficients of single-pass transmembrane proteins measured by FCS in three types of membranes: the native PM, GPMVs, and GUVs (Fig. 1). To address potential artifacts when positioning the confocal spot in the membrane with single-point FCS (spFCS), the diffusion measurements were complemented by circular scanning FCS (csFCS), a technical modification that circumvents some issues related to membranes (Petrášek et al. 2011; Petrov and Schwille 2008). The FCS data analysis is described in considerable detail to emphasize complications that can arise when measuring membrane embedded fluorescent probes. Finally, our results quantify lateral diffusion, and thus, viscosity changes across the different systems and demonstrate a surprisingly pronounced influence of the cytoskeleton on the diffusion of single-pass transmembrane receptors in the plasma membrane of living cells.

## Materials and Methods

### Chemicals

Alexa Fluor 488 succinimidyl ester (‘Alexa488’), the 488 nm excitable lipid analogues 3,3′-dioctadecyloxa-carbocyanine perchlorate (‘DiO’; DiO-C_18_(3)), 3,3′-dilinoleyloxa-carbocyanine perchlorate (‘fast-DiO’; DiO∆^9,12^-C_18_(3) solid), the 543 nm excitable lipid analogue 1,1′-Dioctadecyl-3,3,3′,3′-Tetramethylindo-carbocyanine perchlorate (‘DiI’; DiI-C18(3)), and the 633 nm excitable lipid analogue 1,1′-dioctadecyl-3,3,3′,3′ tetramethylindo-dicarbocyanine, 4-chlorobenzenesulfonate salt (‘DiD’; DiI-C18(5) solid) were purchased from Thermo Fisher Scientific (Darmstadt, Germany). 1,2-dioleoyl-sn-glycero-3-phosphocholine (dioleoylphosphatidylcholine; DOPC), brain sphingomyelin (BSM), and cholesterol (Chol) were purchased from Avanti Polar Lipids (Alabaster, AL, USA) and used without further purification. Ultra-low gelling temperature agarose (type IX-A), paraformaldehyde (PFA), dithiothreitol (DTT), and other chemicals were purchased from Sigma-Aldrich (Munich, Germany). Random labeling of interleukin-4 (IL-4) ligand with amino-reactive Alexa Fluor 647 was performed as described (Weidemann et al. 2011).

### Cells and GPMVs

The stable, HEK293 derived Flp-In cell line expressing the single-pass transmembrane protein NHis-IL4Rαm266-EGFP (N-terminally His-tagged IL-4Rα chain comprised the mature amino acids 1-266 fused to a short-linker ADPPV and a C-terminal EGFP; 540 amino acids in total) and the plasmid pc2-NHis-IL4Rαm241-EGFP-N2, coding for a mutant lacking the intact Box1 motif (N-terminally His-tagged IL-4Rα chain comprised of the mature amino acids 1-241 fused to a short-linker GAGADPPV and a C-terminal EGFP; 518 amino acids in total) are described (Weidemann et al. 2011; Worch et al. 2010). In both constructs, the initial ATG for the EGFP sequence was removed. In the following, we denote NHis-IL4Rαm266-EGFP by ‘H4G266′ and NHis-IL4Rαm241-EGFP by ‘H4G241.’ In H4G241, the EGFP domain locates closer to the transmembrane spanning alpha-helix. Diffusion of H4G266 was measured in the stable Flp-In cell line while H4G241 in transiently transfected HEK293T cells. For lipid analogue mobility measurements, the plasma membrane of HEK293T cells was incubated with fast-DiO (2.5 µg/ml) at 37 °C for one hour. For staining and subsequent measurements, the cells were kept either in Hepes buffered Dulbecco’s modified Eagle’s medium containing 10% serum without phenol red (Life Technologies) or translucent air-buffer designed for live cell observations (150 mM NaCl, 20 mM Hepes pH7.4, 15 mM glucose, 46 mM trehalose, 5.4 mM KCl, 0.85 mM MgSO_4_, 1.7 mM CaCl_2_, and 0.15 mg/ml bovine serum albumin).

GPMVs were produced by PFA/DTT treatment (Baumgart et al. 2007; Tank et al. 1982). Cells were grown to 60–80% confluency in either #1.5 cover slide 8-well chamber slides (Lab-Tek, Thermo Scientific) or 1-well MatTek chamber slides (MatTek Corporation, USA), and washed twice with Hepes pH 7.4 containing 150 mM NaCl and 2 mM CaCl_2_, and bebbing was induced by supplementing 2 mM DTT and 25 mM PFA, followed by overnight incubation at 37 °C. FCS measurements in GPMVs were carried out in the same buffer the next day.

### Nanopatches and Preparation of Giant Liposomes

Nanopatches were produced by differential centrifugation (Swift et al. 2007, 2009). In short, cells were grown to 80–90% confluency in 4 T75 flasks (~40 × 10^6^ cells), washed with ice-cold PBS (phosphate buffered saline), and harvested by scraping. After two more washing steps (pelleting at 1000×g for 2 min), the final suspension was lysed with a tip sonicator (UP50H, Roth, Germany) until the turbid suspension cleared (~1 min). Small soluble fragments of plasma membrane were separated from the large organelles by a centrifugation step (10,000×g for 40 min). The remaining supernatant was again centrifuged (100,000×g for 40 min), and the pellet was resuspended in a small volume of PBS and passed through needles in three steps of increasing gauge (G20, G25, and G27; at least five times each) to disperse the aggregated membrane patches. Finally, the solution was sonicated in a water bath for 10–15 min at room temperature.

Giant unilamellar vesicles (GUVs) were formed from the 1:1 (v/v) mixture of nanopatch solution and DOPC or DOPC/BSM/Chol 1:1:1 small unilamellar vesicles (SUVs) in PBS, concentrated at 10 mg/ml. Physiological conditions of growth were adapted from Hoger et al. (Horger et al. 2009). Droplets of pre-heated 1% (w/w) agarose (Type IX-A) were disposed on a chambered #1.5 cover slide (MatTek) using a metal rod and left for drying on a heater (40 °C). During this step, care was taken to maximize the surface of the agarose film during solvent evaporation. 20 μl of the nanopatches/lipids 1:1 (v/v) mixture was applied on the film and left overnight for drying at 4 °C in a desiccator under vapor pressure of a saturated NaCl solution. The following day, the film was rehydrated with PBS and left for 4 h of swelling.

### Imaging and FCS Measurements

Imaging and single-point fluorescence correlation spectroscopy (spFCS) measurements were performed using the Zeiss LSM 510 or the LSM 780 ConfoCor3 systems, equipped with a C-Apochromat 40x, numerical aperture (NA) 1.2 water immersion objective at room temperature (22.0 ± 1 °C). Images were processed with *ImageJ* applying lookup tables *Green* (EGFP and fast-DiO), *Red* (DiI and DiD), and *Fire* for IL-4-Alexa647.

For spFCS, the focal volume was placed on the top of vesicles according to maximum fluorescence intensity. In the membranes, the fluorescence signal was recorded for at least 15 s and correlated using the built-in electronic hardware correlator (Zeiss Confocor). Autocorrelation functions (ACF) measured for EGFP-tagged receptors in cells, and GPMVs were fitted with a model combining four correlation times: triplet blinking (τ_trp_; varied within 1–10 µs), protonation-dependent blinking for EGFP (τ_nf_; varied within 10–200 µs), a diffusion-related decay for three-dimensional (3D) contributions from the cytoplasm or the interior of the GPMVs (τ_3D_; varied within 200 µs and 2 ms), and a diffusion-related decay for lateral diffusion within the two-dimensional (2D) membrane plane (τ_2D_; larger than 2 ms):

1 $$\begin{aligned} G(\tau ) = G^{T} (\tau ) \cdot G^{D} (\tau ) \hfill \\ G^{T} (\tau ) = \left[ {1 + \frac{{T_{1} }}{{1 - T_{1} }}\exp \left( {\frac{\tau }{{\tau_{\text{trp}} }}} \right)} \right]\left[ {1 + \frac{{T_{2} }}{{1 - T_{2} }}\exp \left( {\frac{\tau }{{\tau_{\text{nf}} }}} \right)} \right] \hfill \\ G^{D} (\tau ) = \frac{1}{N}\left[ {F_{1} \left( {1 + \frac{\tau }{{\tau_{{3{\text{D}}}} }}} \right)^{ - 1} \left( {1 + \frac{\tau }{{S^{2} \tau_{{3{\text{D}}}} }}} \right)^{ - 1/2} + F_{2} \left( {1 + \frac{\tau }{{\tau_{{2{\text{D}}}} }}} \right)^{ - 1} } \right] \hfill \\ \end{aligned}$$

Here *T*_1_, *T*_2_, *F*_1_, and *F*_2_ denote the fractions of the corresponding decays to the overall amplitude G(0) and satisfy *F*_1_ + *F*_2_ = 1. The average number of observed particles *N* = *c V*_eff_ depends on the concentration *c* of fluorescent particles and the size of the detection volume *V*_eff_ that was assumed stretched along *z* by a fixed structural parameter *S* = 5. In physiological GUVs, a 3D diffusion component was not detectable:

2 $$G^{D} (\tau ) = \frac{1}{N}\left( {1 + \frac{\tau }{{\tau_{{2{\text{D}}}} }}} \right)^{ - 1}$$

For fluorescent lipid analogues data were fitted with a single-triplet time (*t*_*t*rp_; varied within 1–20 µs), while the diffusion-related treatment was the same as for the receptors.

3 $$G^{T} (\tau ) = {1 + \frac{T}{1 - T}\left( {\frac{\tau }{{\tau_{\text{trp}} }}} \right)}$$

Cell membranes sometimes move and vesicles suffer from thermally driven shape changes. If distortions of the ACF were minimal, such slow fluctuations were covered by a small fraction (1–10%) of an additional diffusion component for which the correlation time was fixed to 1 s. The focal volume (*w*_0_ ~ 0.2 µm) was calibrated by measuring the diffusion time of Alexa488 in aqueous buffer as a reference for each individual Lab-Tek and evaluating *V*_eff_ = *S*(4π*D* τ_3D_)^3/2^ with the published diffusion coefficient (*D* = 414 μm^2^/s) (Petrov and Schwille 2008). Data analysis was done with *PyCorrFit* (Müller et al. 2014) or self-written *Python* scripts (FCCS in solution).

Circular scanning (csFCS) was performed on a home-built laser scanning microscope using UPLAO 60x W3/IR objective (Olympus) as described (Petrášek et al. 2011). The excitation was provided by a 488 nm laser diode (Sapphire 488-20, Coherent, Santa Clara, USA). The galvanometer scanner was programmed to move the laser focus in a circular trajectory with a radius of *R* = 0.385 µm at a frequency of *f* = 200 Hz. The fluorescence was collected by the objective, transmitted through an appropriate emission filter, and detected by an avalanche photodiode (SPCM-CD2801; PerkinElmer, Wellesley, MA). The recorded photon count sequence was processed by a USB connected correlator device (Flex02-12D, http://correlator.com, Bridgewater, USA) and stored for further software-implemented autocorrelation analysis. The csFCS measurements lasted 100 s. The corresponding autocorrelations curves were fitted to a model function for diffusion with an additional modulation factor for circular scanning:

4 $$G^{D} (\tau ) = G_{0} \left( {1 + \frac{{D_{\tau } }}{{a^{2} }}} \right)\exp \left( { - \frac{{R^{2} \sin (\pi f\tau )}}{{a^{2} + D\tau }}} \right)$$

Here *G*_0_ denotes the amplitude *G*(0), *D* the diffusion coefficient, *a* the width of the focal volume in the *xy* plane, *R* the radius of the scanned circle, and *f* the scanning frequency. Triplet and blinking were neglected. For the IL-4Rα constructs in the plasma membrane and GMPVs, a two-component model was used in the range of 0.01–100 ms. In all other cases, a one-component model was fitted in the time range of 0.01–30 ms. The data analysis for csFCS was performed with Matlab scripts (MathWorks, USA).

## Results

### Receptor Expression in the Plasma Membrane

**Fig. 2:**
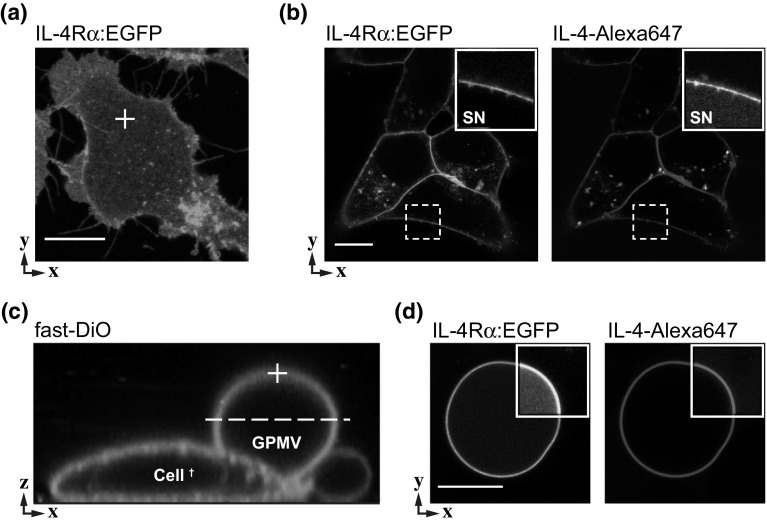
Stable expression of a single-pass transmembrane receptor H4G266 in Flp-In cells. **a** Confocal image of the EGFP-tagged receptor in the bottom plane of the plasma membrane. Typical position for fluorescence correlation spectroscopy (FCS) measurements is indicated (*cross*). **b** Dual-color image of receptor and the Alexa647-labeled ligand IL-4. Magnified view (*inset*) highlights colocalization at the membrane and residual amounts of unbound ligand in the supernatant (SN). **c** Reconstructed image stack (*x*–*z*) of a vesiculated, fast-DiO labeled cell from where giant plasma membrane vesicles (GPMV) were growing; FCS measurements where performed at the pole (cross), confocal imaging at the equatorial plane (*dashed line*). **d** Dual-color images showing that GPMV formation preserves ligand binding capability of the receptor. Note that GPMVs contain visible amounts of EGFP-fragments in their interior (*inset*, contrast enhanced). *Scale bars* 10 μm

We used a stable Flp-In cell line expressing the fluorescent IL-4Rα construct H4G266 as a source for single-pass transmembrane receptors. For yet unknown reasons, surface expression of the truncated receptor H4G266 (Fig. 2a, b) is far better than that of EGFP-tagged full-length receptor (Hintersteiner et al. 2008). Overexpressed full-length receptors transform the morphology of the cells and appear largely retained in perinuclear membrane compartments (Gandhi et al. 2014; Weidemann et al. 2011). In the stable cell line, H4G266 expression is under control of a CMV promoter leading to rather high expression levels of in about 200 receptor molecules per µm^2^ as determined by FCS (Gandhi et al. 2014), however, still exhibiting a quite broad distribution. A homogenous distribution of receptors in the PM is an important prerequisite for accurate diffusion measurements since FCS is inherently very sensitive to aggregation phenomena.

After PFA/DTT treatment, HEK293 cells develop characteristic blebs (GPMVs) that mostly remain attached to the dead cell body (Fig. 2c). Morphologically, GPMVs derived from the stable cell line or transient transfections are indistinguishable. At least in the initial growth phase, the lipid bilayer of GPMVs is in diffusive continuity with the PM. Therefore mobile components, like overexpressed receptors, translocate (partition) continuously into the GPMV membrane (Fig. 2d). We recently found that the degree of partitioning showed surprising variations between different receptor subtypes. For example, IL-4Rα constructs showed superior accumulation within GPMVs as compared to IL-2Rγ variants (Worch et al. 2010).

During a reconstitution procedure, the functionality of the receptor must be maintained. Because hematopoietic cytokine receptors, like the IL-4R subunits, do not possess intrinsic enzymatic activity, we used ligand binding as a readout for structural integrity of the extracellular domain. Applying a fluorescently labeled IL-4 to the supernatant led to specific receptor binding at the surface of cells as well as GPMVs (Fig. 2b, d). Thus, HEK293 cells expressing H4G266 are a suitable model to dissect complex mobility patterns at the plasma membrane from pure lateral diffusion (GPMV) in situ by FCS methods (Fig. 1).

### Plasma Membrane-Derived Nanopatches

**Fig. 3:**
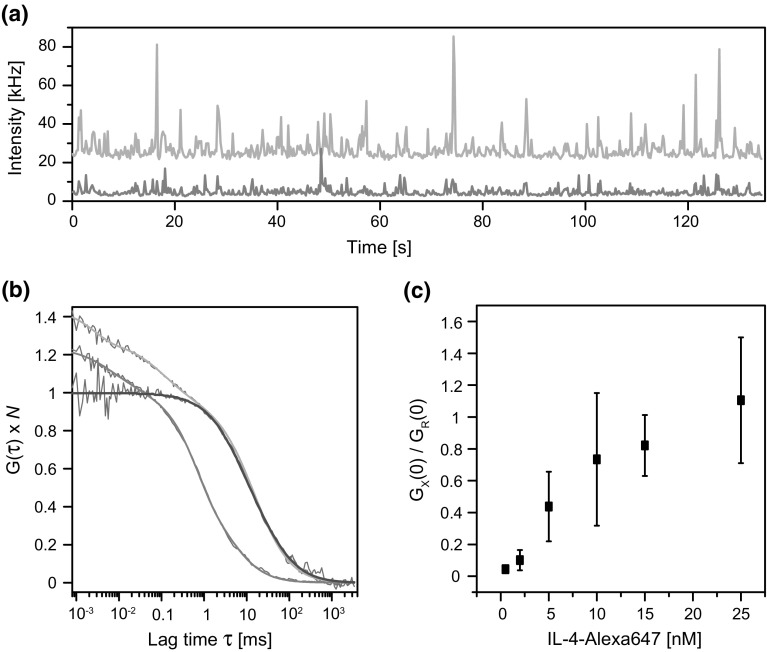
Fluorescence cross-correlation spectroscopy (FCCS) of plasma membrane-derived nanopatches in solution. **a** Intensity traces measured in a solution containing nanopatches (receptor, *green*) and 10 nM IL-4-Alexa647 (ligand, *red*). **b** Corresponding autocorrelation functions (ACF; EGFP, *green*; Alexa647, *red*) and cross-correlation functions (CCF, *blue*) normalized to *N* = 1. The CCF shows that bound ligand co-diffuses with receptor nanopatches. **c** Titration of IL-4-Alexa647 leads to increasing fraction receptor occupied. *Error bars* represent the S.D. of individual measurements (Color figure online)

GPMVs represent still an extremely heterogeneous mixture of lipids and proteins. The only way for reducing the chemical complexity of the membrane environment is to purify the receptor. The purification procedure must avoid high amount of detergents that prevent fusion with liposomes at later stages of the reconstitution into artificial model membranes (Rigaud and Levy 2003). We therefore turned to a recently published method that breaks the PM down into ‘nanopatches.’ The purification relies on cell lysis by sonication, partial centrifugation to isolate the plasma membrane vesicles, followed by a sequence of mechanical shearing through needles of different gauge (Swift et al. 2007). To verify that nanopatches from our stable cell line expressing H4G266 contained intact fluorescent receptors, we performed dual-color FCCS in a solution which was supplemented with nanomolar amounts of the cognate ligand IL-4-Alexa647 (Fig. 3).

Membrane bound fluorescence was already apparent from the intensity time trace in the EGFP channel, which showed numerous ‘spikes,’ indicating fluorescent GFP-tagged receptors diffusing within larger lipid aggregates (Fig. 3a). The broad size distribution reflected by visible spikes could not be narrowed, neither by filtering nor extensive sonication. Correspondingly, fitting of the autocorrelation functions (ACF) required a model containing at least two distinct diffusion components (Fig. 3b). Selecting a subset with rather homogenous traces, we obtained a fast component (*τ*_1_ = 0.19 ± 0.16 ms, *F*_1_ = 42 ± 18%) for blinking and potentially trace amounts of freely diffusing EGFP fragments, and a slower component with 2.5 ± 0.6 ms as the average dwell time of the plasma membrane nanopatches. Applying Stokes-Sutherland-Einstein relationship results in hydrodynamic radii of in about 50 nm in good agreement with literature (67 nm) (Swift et al. 2007). Note the significant time shift between the much faster diffusing unbound protein ligand (red) and the receptor nanopatches (green) (Fig. 3b). Cross-correlating the signals between the two color channels produced a correlation function (CCF, blue) for a single-diffusion component with a mobility corresponding to nanopatches and rather than free protein. Thus, binding sites are confined within nanopatches after purification.

A positive-finite CCF amplitude indicates co-diffusion of ligand with the receptors in nanopatches (normalized to *N* = 1 in Fig. 3b). To exclude potential artifacts associated with non-specific binding we titrated the ligand in a limited concentration range (Fig. 3c). The ratio between the CCF amplitude and ACF amplitude in the red channel is closely related to the fraction receptor occupied (Weidemann et al. 2002). In this particular case, the relationship is non-linear due to multiple labels at both binding partners, the ligand as well as the nanopatches that may bear several copies of receptors. Although saturation was not fully reached, the binding related parameter shows a continuous increase as one would expect for *K*_d_ = 10 nM (Weidemann et al. 2011). Taken together, the FCCS data confirm the structural integrity and accessibility of the receptor’s extracellular domains after purification of plasma membrane-derived nanopatches.

### Reconstitution into Physiological GUVs

**Fig. 4:**
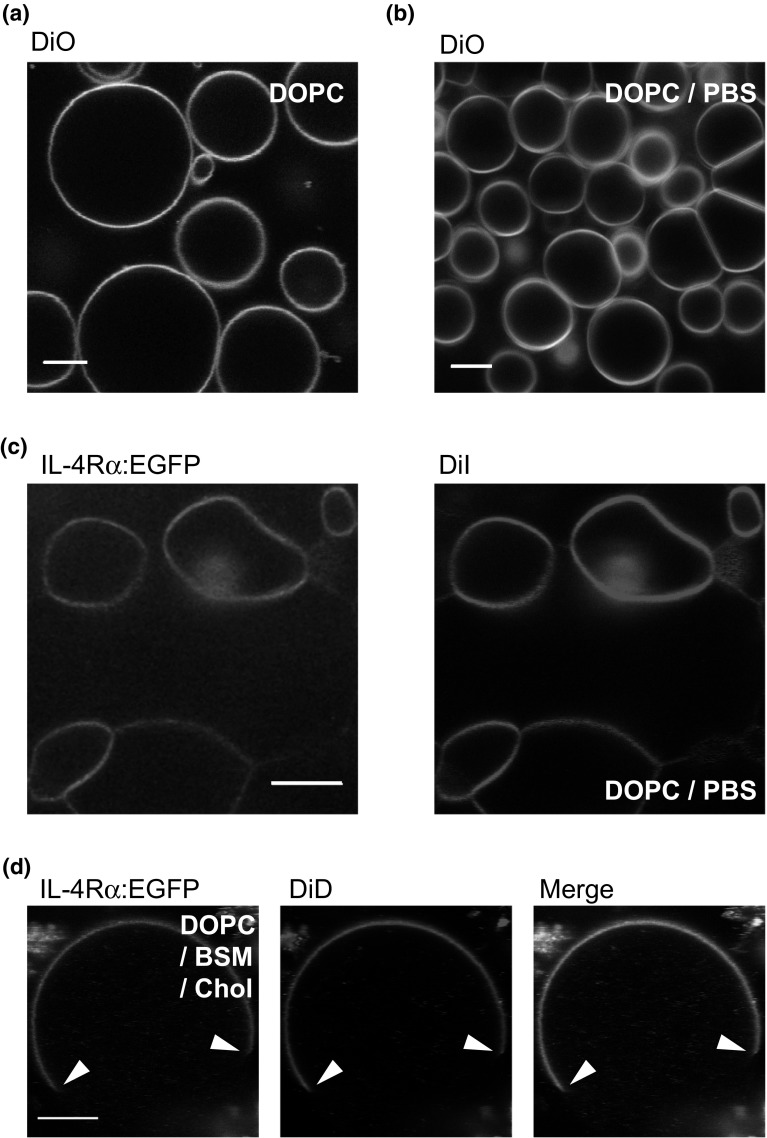
Physiological giant unilamellar vesicles (GUV) produced from nanopatches containing H4G266. **a** ‘Classical’ GUVs composed of DiO doped DOPC using the electro-swelling method in water in comparison to **b** ‘physiological’ GUVs produced by the dehydration method of agarose films. **c** Physiological GUVs produced from nanopatches containing the receptor mixed with DiI-doped DOPC liposomes. **d** Physiological GUVs made from receptor nanopatches and the ‘lipid raft’ mixture DOPC/BSM/Chol doped with DiD. Phase boundaries (*arrow heads*) indicate partitioning of the receptor into the liquid-disordered phase (L_d_). *Scale bars* 10 μm

Due to their small size, nanopatches cannot be used to study lateral diffusion, which requires incorporation of the receptors into larger liposomes. To preserve the protein integrity, we followed the approach of Horger et al., who produced giant liposomes by rehydration of lipids on thin films of dried agarose under physiological conditions (Horger et al. 2009). Receptors are then introduced by mixing nanopatches and SUVs of defined lipid composition (Shaklee et al. 2010). Regarding their size and shape, the agarose rehydrated GUVs in PBS composed of DOPC were similar to conventional GUVs produced by electroswelling (Fig. 4a, b). The membrane of physiological GUVs appeared slightly blurred, which might be and optical effect related to the dehydrated agarose layer on the coverslip. Locally the membranes looked similar, when producing physiological giant liposomes containing nanopatches and receptors, apart from their deformed, non-spherical shape and a tendency to share a quite large cohesive area (Fig. 4c). Importantly, the lateral distribution of the receptor signal within the membrane was homogenous, as for the lipid analogue DiI, indicating diffusive equilibration of the incorporated receptor nanopatches. We also checked that the ligand binding capacity of the receptors was maintained (not shown).

Mixing the native plasma membrane-derived nanopatches with synthetic lipids opens up the possibility of using the lipid mixtures of choice. As an example, we prepared physiological GUVs from nanopatches and DOPC/BSM/Chol (1:1:1), introduced previously as a ‘lipid raft’ model. This lipid mixture segregates into visible, micrometer-sized *L*_o_/*L*_d_ phase domains at room temperature (Fig. 4d). Similarly to many other receptor types, the single-pass transmembrane receptor H4G266 partitioned preferentially into the *L*_d_ phase, following closely the distribution of the fluorescent DiD lipid analogue. Thus, physiological GUVs allow for investigation of receptor–lipid interactions in various environments.

### Lateral Diffusion of Single-Pass Transmembrane Receptors

**Fig. 5:**
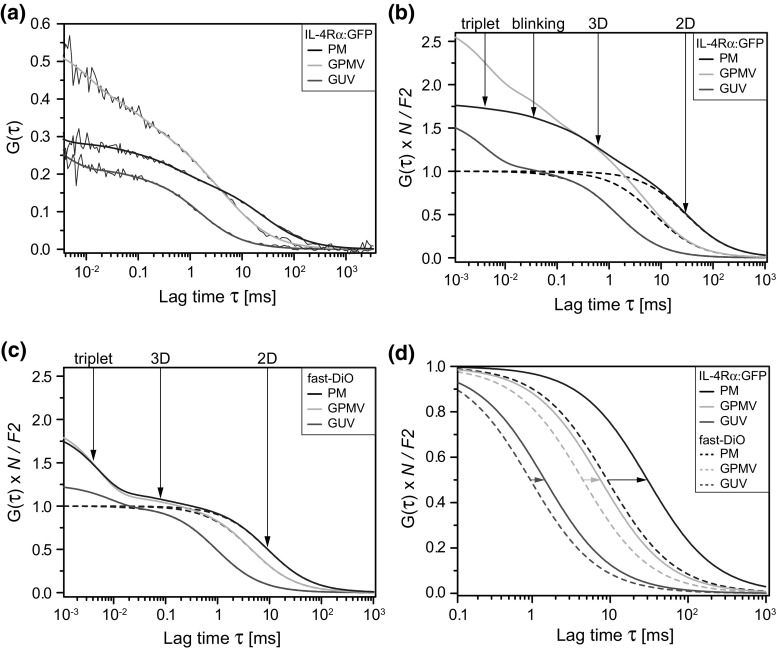
Compilation of single-point fluorescence correlation spectroscopy (spFCS) measurements to determine lateral diffusion in the membrane. **a** Representative ACF examples calculated from 15-s recordings for H4G266 fitted by the appropriate model function (see main text). **b** Calculated autocorrelation functions (ACF) representing average values determined the receptor construct H4G266 and **c** for the lipid analogue fast-DiO in different types of membranes (PM, plasma membrane; GPMV, giant plasma membrane vesicle; GUV, ‘physiological’ giant unilamellar vesicle). ACF have been normalized to *N* = 1; the pure diffusion-related decay is shown separately (*dashed lines*). The amplitude (intercept) of the ACF is larger than 1 due to dark states of the fluorophores. **d** Comparison of diffusion-related decay for both probes in all three types of membranes. The ACF of the single-pass transmembrane receptor is shifted to longer lag times with respect to the lipid (*arrows*) in each system

To explore the effects of membrane architecture and composition, we studied lipid and receptor diffusion by confocal FCS. With spFCS, the detection volume was positioned in the bottom membrane of living cells, at the pole of GPMVs and physiological GUVs (Fig. 1). In cells, the ACF measured for the receptor H4G266 was sufficiently well defined when recording 15 s runs at emission intensities of 2–5 kHz per particle (Fig. 5a). The run time was chosen to balance statistical inaccuracy at longer lag times (>100 ms) against the increasing probability of destructive distortions by global intensity changes (~fluctuations longer than 1 s). The 15 s run time was then kept consistently for all other probes and systems. For cellular measurements, about 30% of the runs had to be discarded; due to the absence of cytoskeleton dynamics, the dropout rate in GPMVs and GUVs was significantly smaller.

Results obtained by spFCS are displayed as calculated ACFs containing the average values obtained by fitting experimental data (Fig. 5b–d; Table 1). For ease of visual comparison, the ACFs have been normalized such that the 2D components (lateral diffusion of lipids and receptors) show the same amplitude (*N*_2D_ = 1). Fitting of the ACF was not straight forward and involved iterative improvements. Averaging the raw data showed that 4 correlation times were necessary to adequately fit curves measured at the PM and in GPMVs. We assumed the following physical processes: (1) short-triplet blinking between 1 and 10 µs a decay that may be contaminated by additional detector after-pulsing, (2) pH-dependent chromophore blinking of EGFP, and (3) two diffusion-related correlation times (Fig. 5b; Table 2). The measurement conditions were such that blinking was not well defined at the PM; therefore, we fixed a mean value from the averaged raw data. Although data averaging introduces a bias toward ACFs with small particle numbers, this mean value agreed well with what we obtained in GPMVs (~50 µs) where blinking was more dominant and could be set free during fitting (Table 1).

**Table 1:** Receptor and lipid dynamics measured by confocal single-point fluorescence correlation spectroscopy (spFCS) and circular scanning (csFCS)

System	Sample	Tau trp [µs]	Tau blink [µs]	Tau 3D [ms]	Dt 2D [µm^2^/s]	Dt 2D^a^ [µm^2^/s]	*n* ^b^
PM	H4G266	2.7 ± 2.7	36^c^	0.6 ± 0.3^d^	0.38 ± 0.15	0.19 ± 0.03	100; 59
	H4G241	3.0 ± 2.6	34^c^	0.5 ± 0.2^d^	0.34 ± 0.12	n. d.	98
	fast-DiO	4.5 ± 1.7	n. a.	0.080^c^	1.3 ± 0.4	0.9 ± 0.3	106; 47
GPMV	H4G266	4.2 ± 3.1	56 ± 28	0.7 ± 0.5^f^	1.5 ± 0.6	1.1 ± 0.3	87; 34
	H4G241	3.5 ± 2.4	46 ± 28	1.0 ± 0.5^f^	1.6 ± 0.5	n. d.	130
	fast-DiO	5.0 ± 3.0	n. a.	0.080^c^	2.5 ± 0.5	2.1 ± 0.4	178; 69
GUV	H4G266	4.0 ± 3.1	39 ± 52	n. a.	8.2 ± 2.0	n. d.	39
	fast-DiO	8.3 ± 5.3	n. a.	n. a.	11.7 ± 1.6	n. d.	24

^a^csFCS

^b^Number of measurements: (spFCS); (csFCS)

^c^Fixed for fitting

^d^Free EGFP fragments in the cytoplasm

^e^Free dye in the supernatant

^f^Free EGFP fragments inside GPMVs

**Table 2:** Fit models

	H4G266	H4G241	Fast-DiO
PM	T–T-3D-2D^a^	T–T-3D-2D^a^	T-3D-2D^c^
GPMV	T–T-3D-2D^b^	T–T-3D-2D^b^	T-3D-2D^b^
GUV	T–T-2D	n. d.	T-2D

^a^Blinking time fixed

^b^Diffusion time of freely diffusing particles fixed

^c^Up to 5% of an additional correlation time fixed to 1 s (global intensity changes)

However, diffusion analysis involved a sub-ms correlation time that was too long to be associated with blinking. For both receptors constructs, H4G266 and H4G241, we obtained at the plasma membrane consistently a fast diffusion time of 0.57 ± 0.27 ms and 0.54 ± 0.20 ms (Table 1) with fractions of in average 38 ± 7 and 33 ± 5%, respectively; stably and transiently expressed receptors showed similar behavior.

Considering a 4–5 times higher viscosity of the cytoplasm as compared to aqueous buffers, these fast diffusion times translate into *D* ≈ 20 µm^2^ s, a mobility that matches well with freely diffusing EGFP in the cytoplasm (Petrášek and Schwille 2008; Weidemann 2014). Such freely diffusing receptor fragments could be associated with a constitutive receptor shedding by metalloproteases (Jung et al. 1999). Indeed, re-examination of images revealed that cells and GPMVs contained a faint intensity background that we interpret as trace amounts of proteolytic EGFP fragments (Fig. 2d, inset). In agreement with confocal images, receptor pulldowns using antibody against GFP showed several bands at lower molecular weight on immunoblots (not shown). Although the concentration of these fragments appeared small, in FCS, these contaminations gain weight due to the fact that (half of) the detection volume is still much larger than the intersecting membrane plane.

At the pole of GPMVs, we obtained for H4G266 a fast diffusion time of 0.74 ± 0.45 ms with a fraction of 40 ± 20% and for H4G241 0.94 ± 0.5 ms with a fraction of 42 ± 17%; thus, both diffusion times and fractions of free EGFP fragments were increased as compared to living cells. We suspect this is caused by a slightly blurred detection volume due to refractive index mismatch in combination with a larger distance from the cover slip. The interior of GPMVs still contains high concentrations of cytoplasmic protein. Assuming a GPMV diameter of 20 µm, the GPMV volume is roughly 4 times larger than a typical cell (1000 µm^3^), which would decrease the refractive index of the cytoplasm from 1.4 to about 1.35 in GPMVs. Theoretical calculations have shown that such an order of magnitude, under typical measurement conditions in solution, can increase the diffusion times by up to a factor of 2 (Enderlein et al. 2005), which agrees remarkably well with our observations.

In GPMVs, the 3D component was slower while the lateral diffusion was increased; therefore, the corresponding correlation times locate closer on the ACF decay (Fig. 5b). To avoid trading between these time regimes, we fitted the curves in two consecutive rounds: first fixing the average blinking time to determine the diffusion time of the EGFP fragments, followed by fixing the average diffusion time of EGFP fragments to finally extract the lateral mobility of the receptors. In physiological GUVs, the situation was simplified in that freely diffusing EGFP fragments were not detectable, and a single 2D diffusion component was sufficient to evaluate the data (Table 2). In GUVs, the triplet and blinking times were in good agreement with previous results. Taken together, the lateral diffusion of receptor constructs (H4G266 and H4G241) gained a factor of 4–5 from the cell surface into GPMVs and another factor of 5 (H4G266) when reconstituted into physiological GUVs (Table 1).

### Lateral Diffusion of Lipids

As for the receptors, spFCS data for lipids are displayed as average ACFs for each experimental system containing the mean values as parameters (Fig. 5c). In contrast to EGFP, the short-time regime (<20 µs) was sufficiently described by a single-dark state corresponding to triplet transitions (Table 2). However, in addition, we detected a fast diffusing species with about 80 µs and a fraction of 19 ± 8% when measuring in the plasma membrane of cells and in GPMVs. In contrast, such a fast 3D component was undetectable in physiological GUVs that have been produced from premixed lipid stocks. We therefore associate this fast component with trace amounts of freely diffusing dye in the supernatant, a contamination that may occur during the ‘extrinsic’ staining procedure with fast-DiO. Taking these factors into account, we found that the lateral diffusion of fast-DiO increased twofold from the PM of living cells into GPMVs and another factor of about 5 into GUVs.

For comparison, we illustrated the relative differences of lateral lipid and receptor mobility within the membrane plane by the corresponding ACF decay for each system (Fig. 5d). The curves representing single-pass transmembrane receptors were consistently shifted to longer lag times and hence indicate slower diffusion than the fluorescent lipid analogue fast-DiO. In vesicles, this difference was smaller than in living cells, where the receptors seemed to experience significant obstructions, even though, the inert EGFP domain is comparably small and locates close to the plasma membrane. Because both the receptors, H4G266 and the shorter version H4G241, show similar diffusion coefficients, we can exclude that the mobility is affected by potential binding of endogenous JAK1. Finally, it is interesting to note that the viscosity in GPMVs is still considerably high and resembles the plasma membrane more than physiological GUVs. Thus, reconstitution of the receptors into physiological GUVs reaches a sufficient dilution step to reproduce mobility levels as expected for pure lipid systems (Weiss et al. 2013).

### Diffusion Measurements by csFCS

**Fig. 6:**
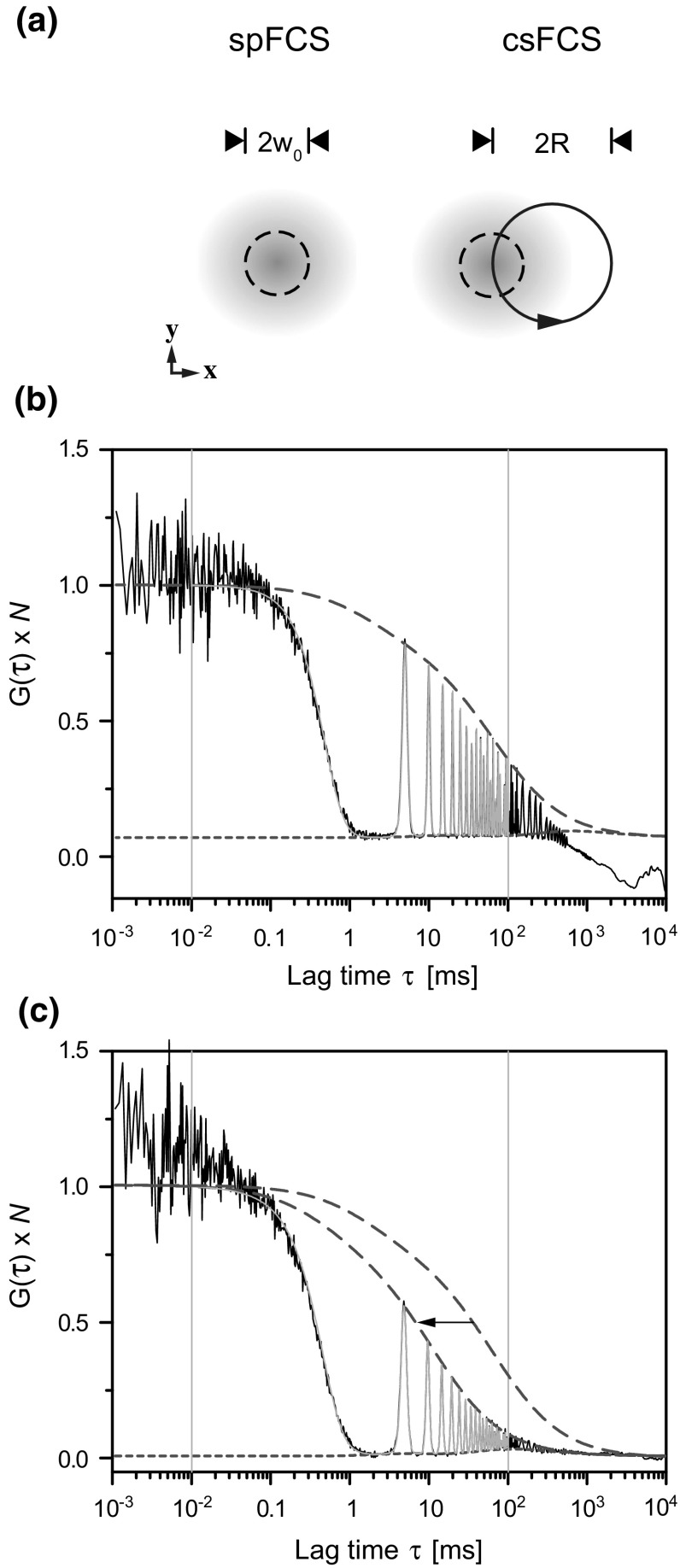
Lateral diffusion measured by circular scanning FCS (csFCS). **a** Schematic representation (*top view*) of the sampled detection area in spFCS and csFCS. The beam waist (*w*_0_ = 0.2 µm) and the scan radius (*R* = 0.385 µm) are drawn to scale. **b** Example csFCS data (*black*) for the single-pass transmembrane receptor (H4G266) at the PM of living cells, normalized to *N* = 1 and fitted with a model function (*red*) containing two diffusion components. The fit oscillated between the upper envelope (*dashed blue line*) and the lower envelope (*dotted blue line*). The data range used for fitting is indicated (*vertical red lines*). **c** Example csFCS data measured for the same cells at the pole of a GPMV. To illustrate the increased mobility (*arrow*), the upper envelope from **b** is shown as a reference (Color figure online)

Diffusion measurements by spFCS are comparative. In spFCS, the dwell time of the particles (diffusion time) depends on the size of the detection volume. Converting diffusion times into diffusion coefficients requires additional calibration measurements of standards, in our case Alexa488 in aqueous buffer. FCS measurements in membranes involve the complication that a horizontal membrane plane of 5–10 nm thickness is cross-sectioning an axially much larger detection volume (*z*_0_ = 1–2 µm). Fluorescence fluctuations arise from a two-dimensional detection area that is smallest at the beam waist but larger when positioning above or below. Thus, with spFCS small inaccuracy in z-positioning may bias diffusion coefficients toward smaller values. In addition, the membrane surface of vesicles or cells can move relative to the objective focus resulting in fluctuations on longer time scales. We therefore complemented our measurements by applying csFCS, a technical extension that ameliorates fluctuation analysis in membranes (Petrášek et al. 2011; Petrášek and Schwille 2008). With csFCS, the confocal spot was positioned the same way as for spFCS. However, during the measurements, it moves on a tiny circular trajectory of defined radius that can be used for internal calibration (Fig. 6a). Because this radius is insensitive to small deviations in *z*, diffusion coefficients and the detection area can be obtained with superior fidelity as independent parameters from the fit.

Circular scanning produced characteristic oscillations with a certain spacing and width in the correlation functions (Fig. 6b, c). Note that the peak maxima over time (upper envelope) follow the shape of a conventional ACF that, for increased mobility, was shifted to correspondingly shorter time regimes (Fig. 6c). Overall, the diffusion coefficients determined for the receptor construct H4G266 and fast-DiO in the plasma membrane of living cells and GPMVs were in good agreement with our spFCS results (Table 1). In contrast to what one might expect related to the positioning issue, csFCS measurements showed a systematic deviation toward smaller diffusion coefficients. The difference between spFCS and csFCS was larger for the receptors than for the lipids and for the receptors, more pronounced at the plasma membrane than in GPMVs. Importantly, the relative mobility changes between the membrane systems were reproduced by both methods: with csFCS, the lipid analogue fast-DiO gained a factor of about 2 from cells to GPMVs, whereas the single-pass transmembrane receptor gained a factor of more than 5.

## Discussion

In this work, we compared the mobility of fluorescent lipid analogues and genetically engineered EGFP-tagged receptors in different model membranes (Fig. 1). Quantification of lateral diffusion by confocal FCS required careful consideration of various additional sources of fluorescence fluctuations including specific photophysical effects of the labels, partial proteolysis, contaminations in bulk solution, and slow membrane movements. Lateral diffusion of single-pass transmembrane and lipids is, respectively, about 10- and 20-fold higher in GUVs than in the plasma membrane (Table 1).

The magnitude of the diffusion coefficients obtained in physiological GUVs is in good agreement with published work that applied FCS approaches. For example, Ramadurai et al. determined that DiD in GUVs composed of DOPC diffuses with a diffusion coefficient of *D* = 9–11 µm^2^/s, whereas membrane proteins of different sizes and model peptides diffused with *D* = 4–5 µm^2^/s (Ramadurai et al. 2009, 2010a, b). A recent comparison of different FCS approaches consistently reproduced *D* = 10 µm^2^/s for DiD in DOPC GUVs (Heinemann et al. 2012). The Enderlein group applied two-focus FCS, an inherently calibrated technique similar to csFCS, and obtained *D* = 8 µm^2^/s for the labeled DOPE lipid and *D* = 2–4 µm^2^/s for multiple-spanning integral membrane proteins in POPC GUVs (Kriegsmann et al. 2009). In black lipid bilayers, the values match even better what we determined in physiological GUVs (Weiss et al. 2013). Therefore, *D* = 8 µm^2^/s of single-pass transmembrane model receptor in DOPC membranes represents a physicochemical upper limit for transmembrane protein diffusion in phospholipid-based biomembranes at room temperature.

Diffusion studies in GPMVs have been less often reported. The order of magnitude of diffusion coefficients is consistent with our own previous work employing perpendicular scanning FCS (Worch et al. 2010) as well as with the fluorescence recovery after photobleaching (FRAP) data reported in the pioneering study on vesiculated muscle cells (Tank et al. 1982). The dramatic difference in viscosity between GPMVs and physiological GUVs suggests not only a larger fraction of integral membrane proteins present in GPMVs, but also that protein–protein interactions and larger agglomerates constitute additional diffusion barriers. Furthermore, although the cytoskeleton is obviously detached, it cannot be excluded that weakly associated, non-filamentous structural membrane components partition into the GPMV interior and reassemble at the inner leaflet of the bilayer. While the mobility in GUVs and GPMVs reflects largely the different molecular dimensions of lipids and the membrane-spanning alpha-helix, the diffusion of receptors in the cellular plasma membrane is disproportionately slower. Indeed, using model membranes it was shown that the dense actin cortex affected the mobility of bulky proteins much stronger than the lipids with small headgroups (Heinemann et al. 2013). Thus, the cytoplasmic EGFP domain seems to sense structural components at the interface between the actin cortex and the plasma membrane.

## Abbreviations

TM: Transmembrane
FCS: Fluorescence correlation spectroscopy
spFCS: Single-point FCS
csFCS: Circular scanning FCS
IL-4: Interleukin-4
IL-4Rα: Alpha chain of Interleukin-4 receptor
GUV: Giant unilamellar vesicle
